# Supplementary material for: Can care staff accurately assess health-related quality of life of care home residents? A secondary analysis of data from the OPERA trial
Source: BMJ Open. 2017 Apr 27;7(4):e012779. doi: 10.1136/bmjopen-2016-012779 (PMC5541407; doi:10.1136/bmjopen-2016-012779)
Supplement: supplementary appendix [file bmjopen-2016-012779supp001.pdf]

Online Appendix A – Model coefficients for self and proxy-reported EQ-5D utilities using imputed data (n = 1060)

Missing data was imputed using multiple imputation with chained equations. Binary variables were imputed using logistic regression and the remaining variables were imputed using predictive mean matching with k nearest neighbours set to 5. Five imputed datasets were created.

Variable	Self-reported	Proxy	Difference (resident-proxy)
**Depression (GDS-15)**	-0.0298* (0.0031)	-0.0081* (0.0027)	-0.0217* (0.0036)
**Cognitive functioning (MMSE)**	-0.0091* (0.0026)	0.0016 (0.0014)	-0.0106* (0.0023)
**Physical functioning (SPPB)**	0.0199* (0.0063)	0.0081 (0.0042)	0.0118 (0.0065)
**Activities of daily living (Barthel Index)**	0.0055* (0.0004)	0.0079* (0.0004)	-0.0025* (0.0006)
**Social engagement (SES)**	-0.0114 (0.0065)	0.0125* (0.0051)	-0.0239* (0.0079)
**Pain Score**	-0.1448* (0.0094)	-0.0385* (0.0101)	-0.1062* (0.0122)
**Dementia**	0.1178* (0.0247)	0.0300 (0.0200)	0.0879* (0.0269)
**Sex**	-0.0374 (0.0221)	0.0073 (0.0168)	-0.0447 (0.0256)
**Age**	0.0020 (0.0015)	0.0012 (0.0010)	0.0008 (0.0016)
**Length of stay**	0.0048 (0.0034)	-0.0012 (0.0032)	0.0060 (0.0045)
**Constant term**	0.5053* (0.1445)	-0.1217 (0.1079)	0.6269* (0.1721)

* indicates statistically significant at the 5% level
Sex: 0 = female, 1 = male
Dementia: 0 = no diagnosis, 1 = diagnosis
